# Dietary fats and cardiometabolic health—from public health to personalised nutrition: ‘One for all’ and ‘all for one’

**DOI:** 10.1111/nbu.12722

**Published:** 2025-01-20

**Authors:** Julie Anne Lovegrove

**Affiliations:** ^1^ Hugh Sinclair Unit of Human Nutrition, Department of Food and Nutrition Sciences University of Reading, Whiteknights Reading UK

**Keywords:** cardiovascular disease, metabolic syndrome, monounsaturated fatty acids, personalised nutrition, polyunsaturated fatty acids, saturated fatty acids

## Abstract

This paper provides a summary of the 2023 British Nutrition Foundation Annual Lecture by Professor Julie Lovegrove. Professor Lovegrove is the head of the Hugh Sinclair Unit of Human Nutrition at the University of Reading. Professor Lovegrove, who was nominated for the BNF Prize for her outstanding contribution to nutritional sciences has published over 300 scientific papers and made a major contribution to establishing the relevance of dietary fat quality in the development and prevention of cardiometabolic disease.

## BACKGROUND

Cardiovascular disease (CVD), once considered to be a degenerative disease of developed, Westernised societies with high rates in the UK, US and Northern Europe, is now common in the global East remaining the greatest cause of death throughout the world (WHO, 2021). In the UK, it is estimated that 480 people will die from heart or circulatory disease every day (BHF, 2024), with those living in deprived areas being over four times more likely to suffer premature CVD than those less deprived (PHE, 2019). Poor diet and lifestyle are major risk factors for the development of CVD, which can be modified to reduce the risk of CVD and its frequently fatal endpoints. ‘Cardiometabolic disease’ is a highly prevalent form of CVD that originates from metabolic abnormalities associated with obesity, most notably central visceral adiposity and deposition of ectopic fat in key metabolic tissues. These tissues (adipose, liver and skeletal muscle) typically develop insulin resistance (failure to respond to insulin) that underlies the generation of adverse metabolic changes (dyslipidaeamia, hyperglycaemia, vascular dysfunction and hypertension), that promote the formation of atherosclerotic plaques in the walls of large arteries. The most common, clinically recognised name for this clustering of CVD risk factors is known as the ‘metabolic syndrome’ which is linked to considerable increased risk of developing CVD and particularly type 2 diabetes.

Diet is a major modifiable risk factor for cardiometabolic diseases, with a reduction in the intake of saturated fatty acids (SFA) being the cornerstone of guidelines for the primary prevention of CVD for over 60 years. The main underlying principle for this guideline is to lower the concentration of blood cholesterol, and specifically low‐density lipoprotein (LDL), a cholesterol‐carrying serum lipoprotein that is causally related to CVD (Ference et al., 2017). In 2019, the UK's Scientific Advisory Committee on Nutrition (SACN, 2019) and the World Health Organization (updated in 2023 (WHO, 2023)) published independent reports based on a review of the totality of evidence from randomised controlled trials and prospective cohort studies relating to dietary SFA and health. Both reports supported the existing guidelines that intakes of SFA should not exceed 10% of total energy and that they should be replaced by unsaturated fatty acids (UFA). The issues of whether SFA should be replaced with UFA and/or carbohydrates, and optimal quality of these replacement macronutrients, have been of great debate and intense study for many decades, and has been a major focus of my own research.

## MY RESEARCH

An overall aim of my research throughout my career has been to provide evidence for the impact of dietary modification in reducing cardiometabolic risk in healthy and ‘at risk’ populations, for the purpose of establishing, translating and implementing optimal dietary requirements for the promotion of cardiometabolic health. This has involved gaining insight into the genetic and metabolic mechanisms that underlie the effects of diet on cardiometabolic risk, and variation in response to diet, for the development of more effective, personalised dietary approaches for the prevention of these diseases. The challenges of implementing and sustaining dietary change have required a diverse range of traditional and novel experimental approaches. While these approaches began by altering the composition of foods to increase their functionality (Lovegrove et al., 1997), they evolved into more ambitious strategies to impact on whole food systems (Lovegrove et al., 2023). These approaches aimed to promote dietary modification for the benefit of human health and minimise adverse effects on the environment to improve overall sustainability. This paper provides an overview of selected areas of my collaborative research from my PhD to ongoing projects.

## OPTIMISING THE QUALITY OF DIETARY FATS FOR IMPROVING CARDIOMETABOLIC HEALTH IN GROUPS SUSCEPTIBLE TO INCREASED CARDIOVASCULAR DISEASE RISK

My research career began with my PhD studies on the effects of maternal cows' milk consumption on subsequent infant allergy, at the University of Surrey (Lovegrove et al., 1993; Lovegrove, Morgan, & Hamptom, 1996). While seemingly unrelated to what would become a lifelong interest in diet and cardiometabolic disease, this early research provided me with invaluable experience in running human intervention studies, which I later transferred to larger trials on dietary fats in humans at risk of cardiometabolic diseases. My PhD also involved the development of a laboratory‐based technique for the identification of milk proteins and antibodies against these allergens (enzyme‐linked immunosorbent assay—ELISA) (Lovegrove, Morgan, & Hamptom, 1996), which I later applied to the measurement of a dietary fat‐transporting blood protein, apolipoprotein B‐48 (Lovegrove, Isherwood, et al., 1996). An impaired capacity to remove fat in the blood after a fat‐containing meal (postprandial lipaemia), had been identified as a source of risk for CVD. It was known that the extent of postprandial lipaemia could be attenuated acutely by altering the composition of fats and carbohydrates in a test meal and reduced over a longer term by similar dietary modifications. Serum apolipoprotein‐B48 proved useful as a novel biomarker for quantifying the extent of postprandial lipaemia, and impact of diet in modifying this CVD risk factor (Lovegrove et al., 1999).

After moving to the Department of Food Biosciences (now Food and Nutrition Sciences) at the University of Reading, my first major research project as a Principal Investigator, examined the potentially favourable effects of dietary long chain n‐3 polyunsaturated fatty acids (LC n‐3 PUFA) on cardiometabolic risk factors in populations from the Indian sub‐continent (specifically Sikh men and women in the Reading area) (Lovegrove et al., 2004). In contrast to dietary SFA, LC n‐3 PUFA were initially linked to lower CVD risk, primarily through their effect in reducing platelet aggregation, which increased clotting time and reduced risk of thrombosis. Pioneering work in this area in the 1980s can be credited to Dr. Hugh Sinclair (Bang et al., 1980), after whom our Human Nutrition Unit was named in honour of his endowment trust being bequeathed to the University of Reading in 1995. Dr. Sinclair, who was keen on self‐experimentation, examined the effect of the typical (of the time) Inuit diet, high in LC n‐3 PUFAs, on his own clotting time over 100 days, with marked reductions (Sinclair, 1984). There was evidence to suggest that a deficiency of dietary LC n‐3 PUFA, and resultant imbalance in the ratio of dietary and tissue n‐6:n‐3 PUFA ratio, could be linked to increased cardiometabolic risk. Although we found supplementation with LC n‐3 PUFA in Sikh and matched Caucasian men and women was associated with reductions in serum triacylglycerol, postprandial lipaemia and increased high density lipoprotein (HDL)—cholesterol (Lovegrove et al., 2004), n‐6 PUFA intake had no impact on these outcomes (Brady et al., 2004).

**FIGURE 1:**
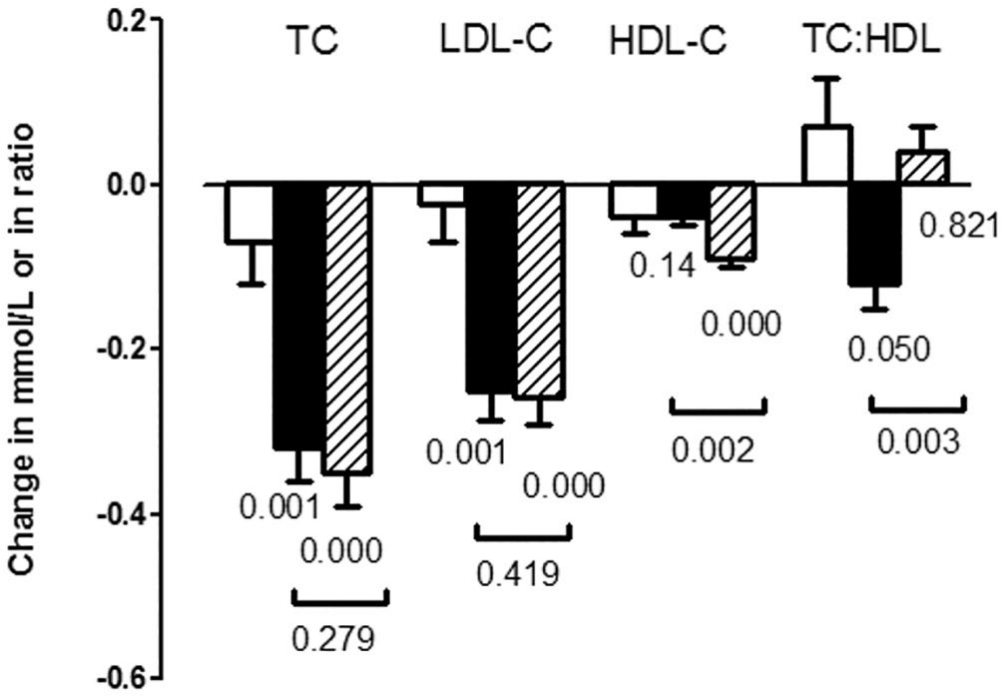
Mean (± SEM) changes in total cholesterol (TC), LDL cholesterol (LDL‐C), HDL cholesterol (HDL‐C), and TC:HDL cholesterol ratio after consumption of diets low in SFA that were high in monounsaturated fatty acids (HM; *n* = 227; filled bars) or low in fat (LF; *n* = 236; hatched bars) compared with a control SFA‐rich diet (*n* = 85; open bars). Values below the bars indicate statistical significance from the SFA diet, and values below horizon bars indicate statistical significance between HM and LF diets (ANCOVA). Data from the RISCK study (Jebb et al., 2010).

There was also evidence at this time to support the idea that the insulin sensitivity in tissues could be increased, and cardiometabolic risk reduced, by replacing SFA with UFA in cell membranes. This idea we tested by replacing dietary SFA with either monounsaturated fatty acids (MUFA) or carbohydrates in two, multi‐centred, randomised controlled trials, the pan‐European *LIPGENE* (*n* = 417) (Tierney et al., 2011) and UK‐based *RISCK* (*n* = 548) (Jebb et al., 2010) studies, in participants either with, or at risk of developing the metabolic syndrome, respectively. Both trials were successful in achieving the dietary exchange of SFA for MUFA and/or carbohydrate over a period of several months, and the replacement of SFA with UFA in cell membranes (Moore et al., 2009; Shaw et al., 2009). The dietary interventions in both trials produced significant reductions in serum total and LDL‐C, after the replacement of SFA with both carbohydrates and MUFA, with greater reduction in the low glycaemic index diet in the *RISCK* study. Benefits to the clinically relevant total:HDL‐C ratio was observed only when SFA was replaced with MUFA, but not carbohydrate (Jebb et al., 2010) (Figure 1). However, no significant effect on insulin sensitivity (IVGTT) was observed in either study (Jebb et al., 2010; Tierney et al., 2011), despite previous evidence of benefit in a healthy group (Vessby et al., 2001).

**FIGURE 2:**
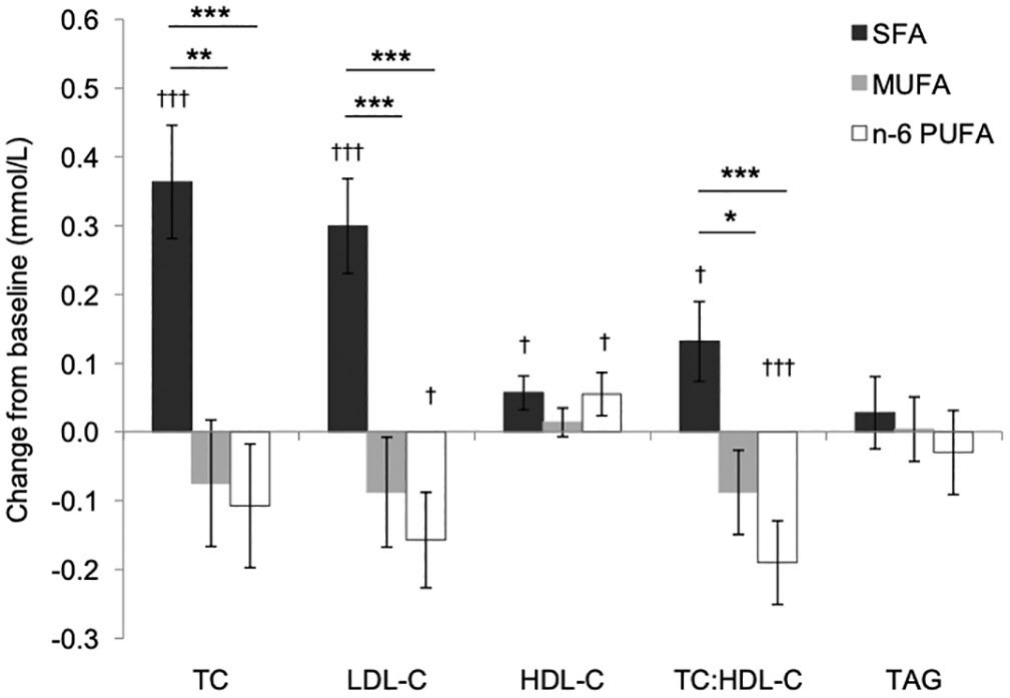
Changes from baseline fasting lipid profile when dietary SFAs were substituted isoenergetically with MUFAs (9.5%TE) or n–6 PUFAs (9.6%TE) for 16 weeks. Data are means (± SEMs), *n* = 58–62 per diet group. Overall diet effects were statistically significant for total cholesterol (TC), low‐density lipoprotein (LDL) cholesterol, and TC:HDL cholesterol ratio (*p* ≤ 0.001). Post hoc analysis identified significant between‐group differences (*p ≤ 0.05, **p ≤ 0.01, ***p ≤ 0.001). TAG, triacylglycerol; %TE, percentage of total energy. Data from the DIVAS study (Vafeiadou et al., 2015).

While *LIPGENE* and *RISCK* helped establish evidence for the overall benefit to cardiometabolic health of replacing SFA with UFA, rather than carbohydrates, the important question remained, should SFA be replaced with MUFA or PUFA? This was addressed in the *DIVAS* study, a randomised controlled study with outcome measures of more novel CVD risk (including vascular dysfunction: flow‐mediated dilatation, arterial stiffness and microvascular function) and classic lipid risk factors in men and women (*n* = 150) at increased cardiometabolic risk. The replacement of SFA with MUFA or PUFA in *DIVAS* had no significant effect on these measures of vascular function. The MUFA and PUFA diets produced significant reductions in serum LDL‐cholesterol (Figure 2) but only MUFA was associated with a significant attenuation of the increase in night‐time blood pressure (an independent risk factor for CVD) produced by a high SFA diet together with a serum biomarker of vascular dysfunction, E‐Selectin (Vafeiadou et al., 2015). In a further investigation of mechanisms linked to vascular dysfunction, replacement of SFA with MUFA and PUFA was shown to be associated with significant decreases in microparticles, which represent a marker of damage to the lining of blood vessels and increased CVD risk. Conversely, only MUFA was accompanied by a significant increase in endothelial progenitor cells, which repair damaged lining of blood vessels and are associated with reduced CVD risk (Weech et al., 2018). *DIVAS* therefore supplied evidence in support of dietary MUFA as an effective substitute for SFA with some benefits, over PUFA, on vascular CVD risk factors.

## PERSONALISED NUTRITION

Approximately 99% of the human genome is identical, but like other species, we show vast differences in our physical appearance, behaviour, and less obviously, physiological responses to our environment. The latter includes marked inter‐individual variation in metabolic responses to diet. For example, there are dramatic variations in the response of blood cholesterol to changes in the intake of SFA between individuals. This variation in response, primarily in serum LDL‐cholesterol, has been well documented and was evident in the *RISCK* and *DIVAS* studies (Griffin et al., 2021) and has major implications for increasing the efficacy of diet in reducing CVD risk by the tailoring of advice to responsive and unresponsive groups.

Of numerous genetic variants associated with serum LDL‐C, polymorphism in the apolipoprotein E gene, and more specifically, carriage of the E4 allele, is one of the most common and well‐documented genetic variants linked to elevated serum LDL‐C and greater sensitivity to the LDL‐raising effect of SFA. In the *SATgenε* study, carriage of the E4 allele, in overweight men who had been prospectively genotyped for the apo‐E polymorphism, were found to be less sensitive to the serum triacylglycerol and C‐reactive protein (a marker of chronic inflammation, a risk factor of cardiometabolic disease)‐lowering effect of the LC n‐3 PUFA, docosahexaenoic acid (DHA) (Carvalho‐Wells et al., 2012). This suggested, that in addition to being predisposed to higher CVD risk related to elevated LDL‐C and SFA, carriers of apo E4 may also require a higher intake of DHA, for its benefits to cardiometabolic health.

Despite the identification of other genetic loci that were shown to be of relevance to dietary responsiveness and cardiometabolic risk in the *RISCK* and *LIPGENE* studies (AlSaleh et al., 2011; Alsaleh, Frost, et al., 2011; Garcia‐Rios et al., 2011; Perez‐Martinez et al., 2011), it was accepted that common variation in the metabolic response to diet involves a small degree of heterogeneity in a very large number of genes. This limits the application of single genetic polymorphisms in predicting the responsiveness to diet, the alternative approach of measuring metabolic traits as outcomes of this genetic variation offered a novel supporting strategy. This led to the popularity of the ‐omics techniques.

**FIGURE 3:**
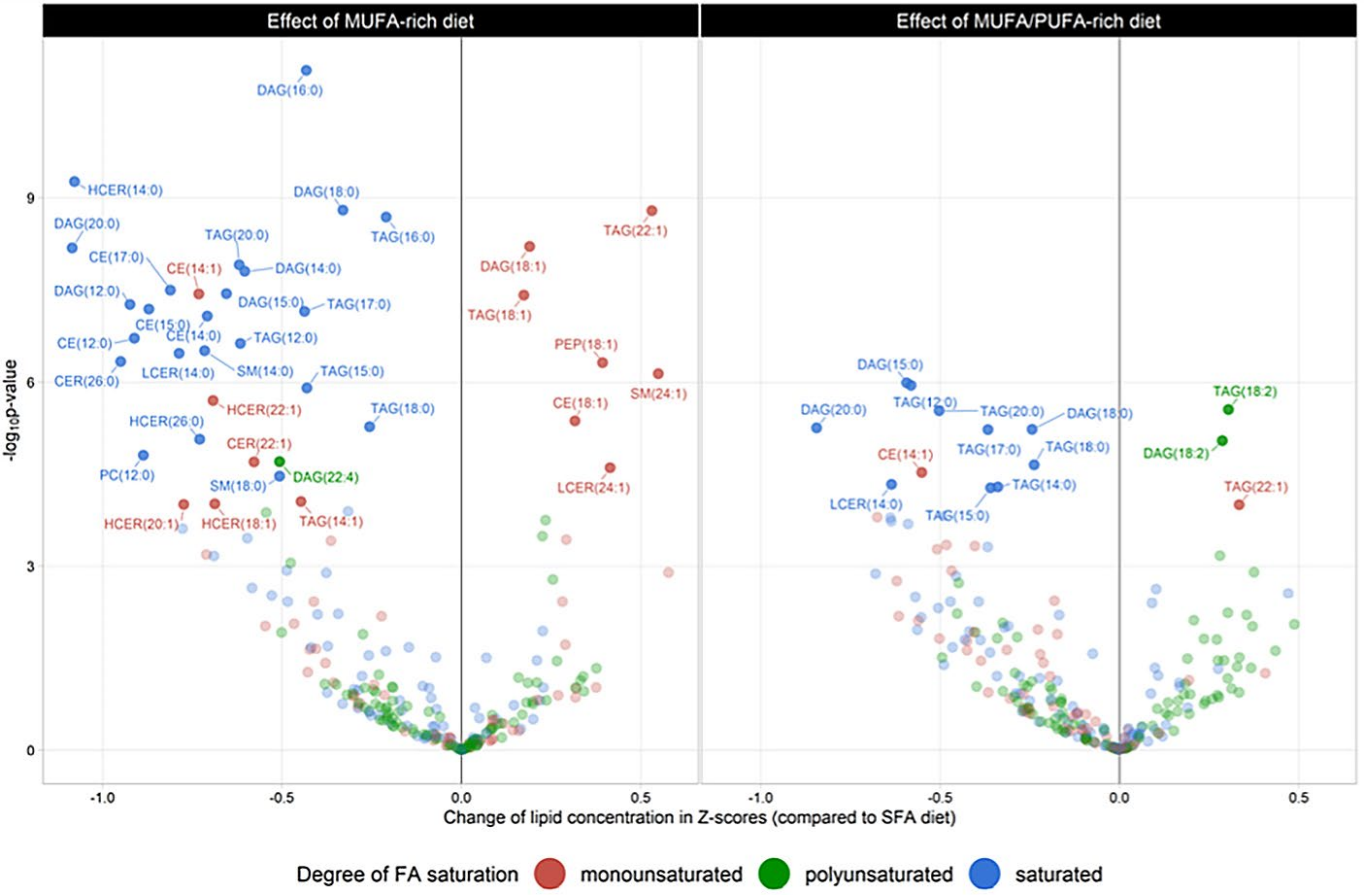
Effect of MUFA‐rich and MUFA/PUFA–rich dietary interventions compared with a SFA‐rich diet on plasma lipid metabolites identified in lipidome‐wide screening among participants from the DIVAS study (*n* = 113). Unlabelled data points represent within‐class fatty acid concentrations not significantly affected by the DIVAS dietary intervention after Bonferroni correction (*p* < 0.05). CE, cholesteryl ester; CER, ceramide; DAG, diacylglycerol; DCER, dihydroceramide; HCER, hexosylceramide; LCER, lactosylceramide; LPC, lysophosphatidylcholine; LPE, lysophosphatidylethanolamine; MAG, monoacylglycerols; PC, phosphatidylcholine; PE, phosphatidylethanolamine; PEO, phosphatidylethanolamine ether; PEP, phosphatidylethanolamine plasmalogen; PI, phosphatidylinositol; SM, sphingomyelin. Data from the FAME study (Sellem et al., 2023).

To gain further insight into how variation in blood lipids relates to diet and cardiometabolic risk, we undertook a study of the serum lipidome in collaboration with colleagues at the University of Potsdam. A deep lipidomic analysis of serum from our *DIVAS* study was used to construct a multi‐lipid score (MLS), summarising the effects of replacing SFA with UFA on 45 lipid metabolite concentrations. A difference in the MLS, reflecting better dietary fat quality, was associated with a significant 32% reduction in the incidence of cardiovascular disease and 26% reduction in type 2 diabetes in the *EPIC‐Potsdam* cohort (Eichelmann et al., 2024). Furthermore, specific lipids associated with cardiometabolic disease risk were found to be beneficially changed by a dietary fat intervention further supporting the substitution of dietary SFA with UFA as a potential tool for primary disease prevention (Eichelmann et al., 2022; Sellem et al., 2023) (Figure 3).

**FIGURE 4:**
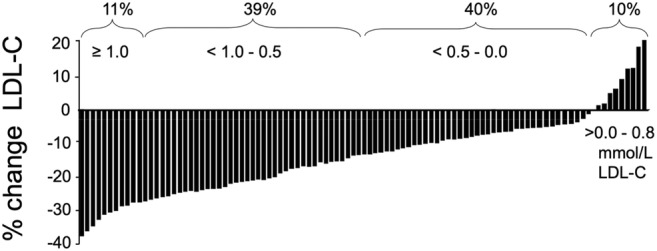
Individual changes in serum low‐density lipoprotein (LDL) cholesterol (mmol/L) in response to replacing saturated fatty acids (SFA) with unsaturated fatty acids (UFA). Each bar represents an individual participant's (*n* = 109 adult males) change in LDL cholesterol (Δ LDL‐C) after replacing dietary SFA (higher‐SFA/ lower‐UFA diet) with UFA (lower‐SFA/higher‐UFA diet) for 4 week. Data from the RISSCI study adapted from (Koutsos et al., 2024).

The more recent *RISSCI‐1* study reported a similar variation in serum LDL‐C in response to the removal and replacement of SFA as seen in *RISCK* and *DIVAS* (Koutsos et al., 2024). The RISSCI‐1 study was designed to investigate the genetic and metabolic origins of variation in serum by reproducing the effects of national dietary guidelines. This was achieved by replicating a transition from a higher to a lower SFA intake (<10%E) with SFA being replaced by UFA (Sellem et al., 2022). While the dietary intervention lowered serum LDL‐C in the majority of participants (90%) the mean response of which would be considered clinically significant (0.5–1 mmol), it produced responses in LDL‐C ranging from approximately −38% to +19% (Koutsos et al., 2024) (Figure 4). While variation in dietary compliance can never be ruled out as a contributor to variation in serum LDL‐C, *RISSCI‐1* reported excellent adherence to the diets (Sellem et al., 2022). This implicates innate genetic and metabolic differences between individuals with the potential to influence the absorption, subsequent transport and fate of dietary SFA, and other determinants of cholesterol homeostasis.

The principal mechanism by which dietary SFA raises serum LDL‐C is by downregulating the transcription of the LDL receptor gene, reducing the number and activity of cellular LDL receptors, and in the process, increasing intracellular cholesterol synthesis. The absorption of cholesterol originating from the diet and bile in the gut is reciprocally related to these events and would be expected to increase if the intake of SFA and cholesterol synthesis are reduced, and LDL‐receptor activity is increased. This is consistent with our findings in the *RISSCI‐1* study. Three separate biomarkers of cholesterol absorption increased following the removal and replacement of SFA, together with the expression of the LDL‐receptor gene in circulating peripheral blood mononuclear cells (Koutsos et al., 2024). Although consistent with the overall reduction in serum LDL‐C, these findings did not account for the substantial variation in serum LDL‐C. This was explained by only two variables, the initial concentration of serum LDL‐C before the dietary intervention, and the decrease in dietary SFA on transitioning from the high to lower SFA (high UFA) diets (Koutsos et al., 2024). Underlying mechanisms for this individual variation in LDL‐C response to dietary fat intake were investigated in the follow‐up, *RISSCI‐2* study. This study used an isotopically labelled SFA to trace the absorption, distribution, and elimination of this dietary fat, deep phenotyping of the gut microbiota, and NMR metabolomics to identify simple biomarkers of more complicated metabolic traits with analysis almost completed.

**FIGURE 5:**
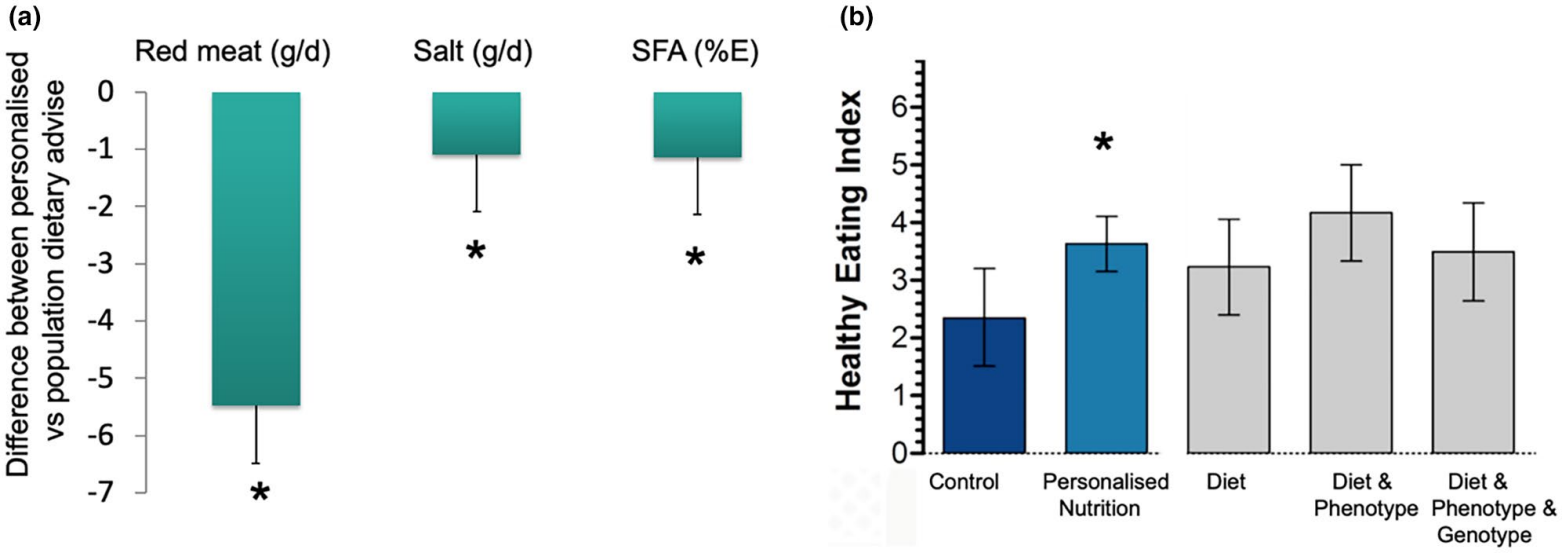
(a) Mean (± SEMs) of difference in red meat * (*p* = 0.046), salt * (*p* = 0.008) and saturated fatty acids * (SFA) (*p* = 0.0001) intakes between the personalised dietary advice and control (general public health advice) groups. (b) Mean (± SEMs) change in Healthy Eating Index (a measure of diet quality) in the control, personalised dietary advice group combined * (*p* = 0.01), and personalised dietary advice based on diet; diet and phenotype; and diet, phenotype and genotype. Data from the Food4Me study adapted from (Celis‐Morales et al., 2017).

An important aspect of personalising dietary advice is to determine what motivates people to change their diet and lifestyle, in addition to identifying what specific advice is beneficial on an individual/group level. *Food4Me* was a multi‐centred, EU‐funded project that aimed to address two questions. Firstly, ‘does personalisation of dietary advice motivate a healthier diet compared with population dietary guidance?’ and secondly, ‘is personalisation based on phenotypic or genotypic information more effective in motivating healthy choices, than personalisation based on diet alone?’ *Food4Me* was based on the online delivery of food‐based personalised dietary advice using a validated food frequency questionnaire compared with dietary guidance (Fallaize et al., 2014). After screening 5500 participants in Europe, 1700 participants were assigned to one of four groups: (i) general dietary advice; personalised nutrition based on (ii) diet; (iii) diet and profile of blood biomarkers of CVD risk (phenotype) (iv) diet, blood and genetic biomarkers of CVD risk. Personalised dietary advice was associated with greater motivation to change diet than general dietary advice alone (Celis‐Morales et al., 2017). More specifically, there was a significantly greater reduction in the intake of SFA, processed red meat and salt and improved overall diet quality in those receiving personalised compared to general public health recommendations (Figure 5). Despite these apparent benefits of personalised advice, motivation to change diet was not influenced by prior knowledge of phenotype or genotype in the *Food4Me* study (Celis‐Morales et al., 2017). These data were further supported by the application of our online personalised nutrition advice tool (eNutri) offering personalised food‐based dietary advice according to dietary intake assessed by an updated, validated and culturally inclusive food frequency questionnaire and comparing this to a diet quality index (Franco et al., 2017) in UK (Zenun Franco et al., 2022) and German populations (Blaurock et al., 2021).

To provide effective dietary advice based on an individual's diet, accurate dietary assessment is essential and remains a key challenge for nutrition science, as most of the tools are subjective, requiring the individual to report their food and drink intake either prospectively (e.g. diet diaries) or retrospectively (e.g. 24‐h recalls, food frequency questionnaires) which is compared with dietary databases to assess nutrient intake. Such methods can lead to misreporting and inaccurate dietary assessment. The ongoing *SODIAT* study was designed to overcome these limitations by developing an objective dietary assessment tool that is independent of subjective input. An aim of *SODIAT* is to increase the accuracy and precision of diet assessment and monitoring to improve public dietary advice and health, by developing and applying more objective measures of dietary intake by using serum/urine biomarkers and food capture on camera (Bobokhidze et al., 2024).

## NOVEL APPROACHES FOR TRANSITION TO MORE SUSTAINABLE DIETS TO IMPROVE PUBLIC AND ENVIRONMENTAL HEALTH

For the outcomes of research to be transformative in changing diet and lifestyle behaviours and achieve a significant reduction in CVD risk, our experimental approaches needed to move beyond functional foods (Lovegrove et al., 1997) and food exchange models (Moore et al., 2009; Sellem et al., 2023; Shaw et al., 2009), and consider sustainable diets for both human and planetary health (Lovegrove et al., 2023). Supporting diet and lifestyle behaviours for long‐term benefits to human and planetary health would require changing food systems, with impacts on food production, distribution (agriculture, industry, commerce) and the environment.

**FIGURE 6:**
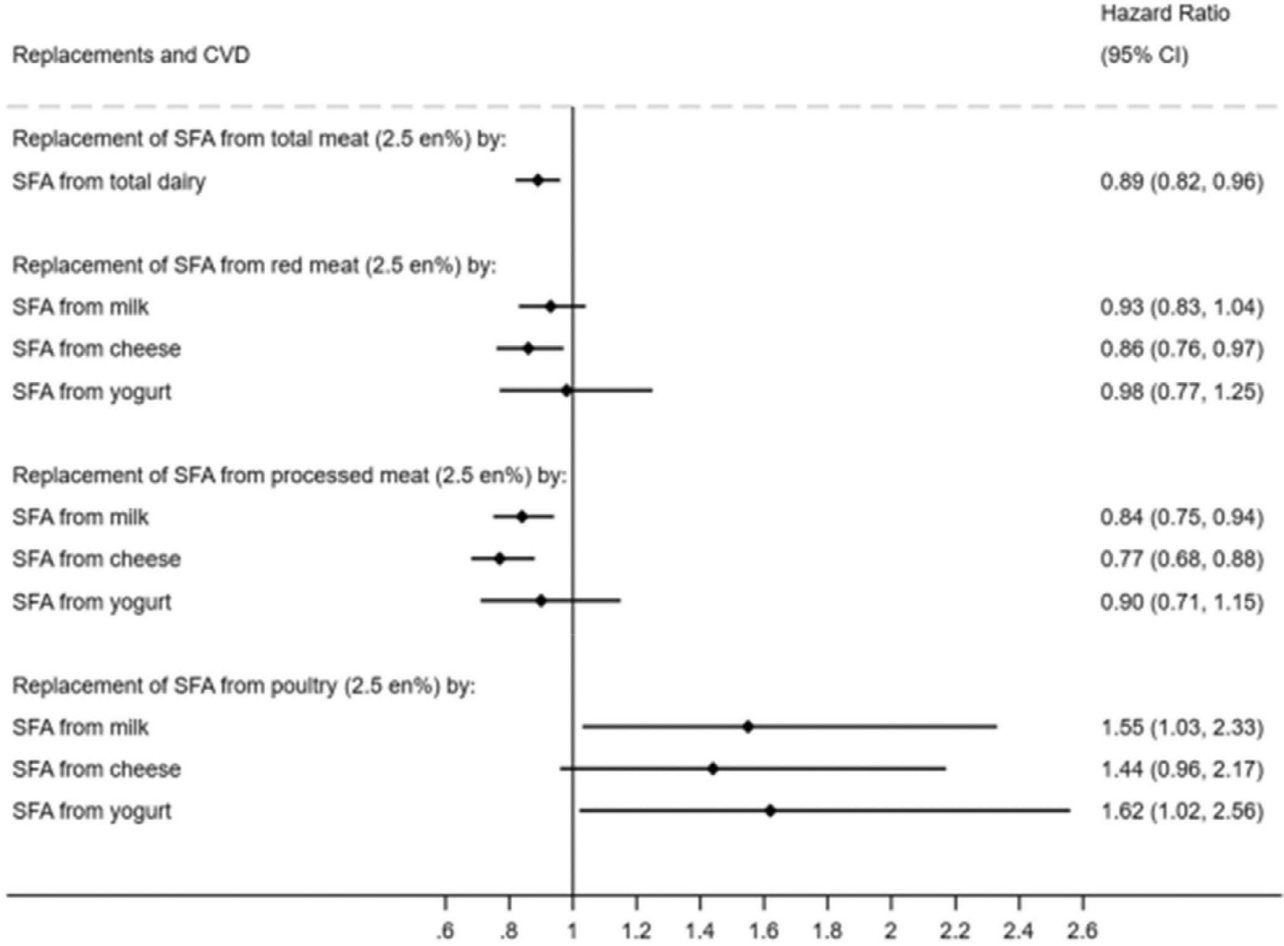
Modelled replacement of saturated fatty acids (SFA) from total, red, processed, or poultry meat (2.5% of total energy) by the equivalent from total and types of dairy in relation to incident CVD: The EPIC‐Norfolk Study (*n* = 21 841). No. of CVD cases: 5902 over person‐years of 395854.5. CVD, cardiovascular disease; en%, percentage of total energy intake; EPIC, European Prospective Investigation into Cancer and Nutrition (Vogtschmidt et al., 2024).

Dairy products are nutrient‐dense, relatively low cost and staple foods in the UK, consumed by the majority of the population, including residents in deprived areas. Although dairy makes the greatest contribution to SFA in the UK and in many European diets, we and others have shown that dairy‐rich diets are associated with a greater diet quality (Hobbs et al., 2020) and lower CVD risk (Guo et al., 2017), and are associated with benefits to CVD risk when replacing meat, another food high in SFA (Vogtschmidt et al., 2024) (Figure 6). The apparent conflict between the effects of high SFA and an increase in CVD risk, compared with higher dairy and lower CVD risk can be explained by other components in dairy counteracting the adverse effects of SFA on such CVD risk factors as high blood pressure and cholesterol. These other components may include minerals, bioactive peptides, and the protein profile that can lower blood pressure and improve vascular function and reduce SFA absorption (Fekete et al., 2016, 2018). Optimising the composition of milk for cardiovascular health will therefore require reducing its SFA content while maintaining its profile of these other components. This can be achieved by the common practice of skimming the fat, but SFA re‐enters the human food chain as cream or butter.

We addressed this issue using an alternative food chain approach in the *RESET* study (Vasilopoulou et al., 2020). When cows were fed high oleic (MUFA‐rich) sunflower oil for 4 weeks, their milk contained significantly lower SFA and higher MUFA (Markey et al., 2017). Although the milk, cheese and butter made from this milk had a more optimum fatty acid profile, it was important to determine the effects of its consumption on human health. A double‐blind, crossover, randomly controlled study in which 63 men and women at risk of CVD consumed a high dairy diet containing conventional or fat‐modified milk, butter and cheese for 12 weeks was performed to address this issue. Increases in serum LDL‐cholesterol and measures of vascular dysfunction on the control diet were significantly attenuated by the fat‐modified dairy products (Vasilopoulou et al., 2020). This provided further evidence that although SFA in the conventional dairy led to increased CVD risk factors, exchange for MUFA attenuated these negative effects. If applied county‐wide, this food chain approach to modifying the fat profile of dairy foods could lead to an estimated removal of 86 000 tonnes of SFA from the food chain annually. At the same time, consideration of the environmental impacts of foods is key. Dairy‐containing diets are reported to have a higher environmental impact than plant‐based diets (Hobbs et al., 2020). However, research at the University of Reading has shown that the carbon emissions from cows in the form of methane can be reduced by approximately 25% by feeding cows unsaturated fatty acid‐rich oils (Kliem et al., 2019). A food chain approach to lower SFA intake can therefore also exert a positive impact on the environment by reducing the carbon footprint of dairy products.

Transition to a more plant‐based diet is an effective strategy to support a more sustainable diet key to the National Food Strategy (Dimbleby, 2022) and is supported by the Eatwell Guide (PHE, 2016). Pulses are nutrient dense (rich in protein, fibre and micronutrients, such as iron and zinc) and can fix atmospheric nitrogen (due to symbiosis with Rhizobium bacteria in root nodules), thus reducing the requirement for nitrogen‐rich fertilisers, and can be sustainably grown in the UK. Their consumption is associated with beneficial health effects including blood lipids, glycaemic control, inflammatory status, oxidative stress and gut microbiota (Ferreira et al., 2021). Pulses count towards one portion (80 g) of our 5 A DAY fruit and vegetable intake, yet, despite the environmental, nutritional and health benefits, pulses are poorly consumed with an estimated UK intake of 15 and 10 g/day for adults and children, respectively (Kaimila et al., 2023; Olotu et al., 2023). *Raising the Pulse* is an ongoing collaborative multi‐disciplinary study that aims to transform UK food systems to increase the consumption of pulses in all groups within the population. In addition to encouraging pulse intake, reformulation of staple foods such as bread (purchased by 96% population), by, for example, the substitution of nutrient‐poor white flour with nutrient‐dense faba bean flour is a novel approach to improve the nutrient composition of bread and population diets, without the need for diet changes (Lovegrove et al., 2023). We anticipate the *Raising the Pulse* bread will achieve good market penetration, more diverse cropping, lower fertiliser use, lower glycaemic index and ultimately a reduction of CVD risk factors in consumers. It will also reduce the need for imported soy (used as an improver in all white bread). The overall impact of this approach on land use, human and environmental health, costs and consumer acceptability are other considerations to be assessed.

## CONCLUSION

Evidence‐based dietary guidelines are essential to public health, but the translation of recommendations will require the availability of healthful foods and a sustainable diet for all population groups. For dietary fats, there is evidence to demonstrate that the replacement of SFA with UFA benefits cardiometabolic health. However, behavioural and physiological responses to such advice are variable and depend on metabolic, genotypic and environmental factors and the nature of SFA‐containing foods. Personalising dietary advice has been shown to improve the effectiveness of recommendations and enhance dietary behaviour changes. Moving forward, future challenges include the provision of diets that are assessable, acceptable, nutrient‐dense, affordable and palatable for all groups of the population, an ambition that will require collaboration with stakeholders in all sectors of the food system.

## FUNDING INFORMATION

None.

## CONFLICT OF INTEREST STATEMENT

The author declares no conflict of interest.

## Data Availability

Data available on reasonable request from the author.
